# Synthesis and anticancer activity of the derivatives of marine compound rhizochalin in castration resistant prostate cancer

**DOI:** 10.18632/oncotarget.24764

**Published:** 2018-03-30

**Authors:** Sergey A. Dyshlovoy, Katharina Otte, Kseniya M. Tabakmakher, Jessica Hauschild, Tatyana N. Makarieva, Larisa K. Shubina, Sergey N. Fedorov, Carsten Bokemeyer, Valentin A. Stonik, Gunhild von Amsberg

**Affiliations:** ^1^ Laboratory of Experimental Oncology, University Medical Center Hamburg-Eppendorf, Department of Oncology, Haematology and Bone Marrow Transplantation, Section Pneumology, Hubertus Wald-Tumorzentrum, Hamburg, Germany; ^2^ Laboratory of Marine Natural Products Chemistry, G.B. Elyakov Pacific Institute of Bioorganic Chemistry, Vladivostok, Russian Federation; ^3^ School of Natural Sciences, Far Eastern Federal University, Vladivostok, Russian Federation

**Keywords:** rhizochalin, castration resistant prostate cancer, androgen receptor, AR-V7, apoptosis

## Abstract

Development of resistance to standard therapies complicates treatment of advanced prostate cancer. Alternative splicing variants of the androgen receptor (AR), e.g. AR-V7 can mediate resistance to AR-targeting substances abiraterone and enzalutamide. Semi-synthetic marine natural compound rhizochalinin decreases the expression of AR-V7 in human castration-resistant prostate cancer cells and thus resensitizes cells to enzalutamide.

In the current study, we modified the structure of rhizochalin in order to determine structure-activity relationships (SAR) and optimize anticancer properties. Thus, we synthesized new 18-hydroxy- and 18-aminorhizochalins and its aglycones. All compounds exhibited anticancer properties in human castration-resistant prostate cancer cells, induced apoptosis and G2/M cell cycle arrest, and were capable of autophagy inhibition. SAR analysis showed an increase of pro-apoptotic activity in the row 18-amino < 18-hydroxy < 18-keto derivatives. In general, aglycones were more cytotoxic compared to glycosides. The sugar elimination was critical for the ability to suppress AR-signaling. Rhizochalinin (2) and 18-hydroxyrhizochalinin (4) were identified as the most promising derivatives and are promoted for further development.

## INTRODUCTION

Androgen receptor (AR) signaling is crucial for the growth and development of normal and malignant prostate cells. In fact, androgen deprivation therapy (ADT) with GnRH agonists and antagonists is an essential step in prostate cancer treatment [1]. However, ADT eventually fails twelve to eighteen months after treatment initiation leading to the development of castration-resistant prostate cancer (CRPC) [1]. Second generation AR-targeting drugs enzalutamide and abiraterone are approved for the treatment of metastatic CRPC before and after chemotherapy with docetaxel. While enzalutamide blocks the ligand-binding domain of AR and thus prevents the binding of its natural androgen ligands [2], abiraterone inhibits cytochrome P450 17A1, which is required for adrenal and intratumoral androgen production, and therefore suppresses the androgen level in the tumor [2]. However, primary and secondary resistance to these drugs have been observed [3]. Indeed, response to the second AR-targeted drug in CRPC is decreased and a decline of progression-free survival has been reported with each additional treatment line [4].

A potential mechanism of resistance to enzalutamide and abiraterone is the expression of alternative mRNA splicing variants of the AR [5], e.g. AR-V7 [6, 7]. In contrast to native full-length AR (AR-FL), the AR-V7 lacks the C-terminal ligand binding-domain, which prevents the binding of androgens. At the same time, AR-V7 exerts its transcriptional functions without androgen binding resulting in a constitutive activation of the AR transcriptional program. This ultimately results in enhanced prostate cancer cell proliferation and survival despite enzalutamide and abiraterone treatment [2, 6].

Pro-survival autophagy is another mechanism of drug resistance in human prostate cancer cells [8, 9]. It helps cancer cells to overcome stress conditions, such as chemo- and radiotherapy [10]. In contrast, inhibitors of autophagy may increase anticancer therapy outcome [10].

**Figure 1:**
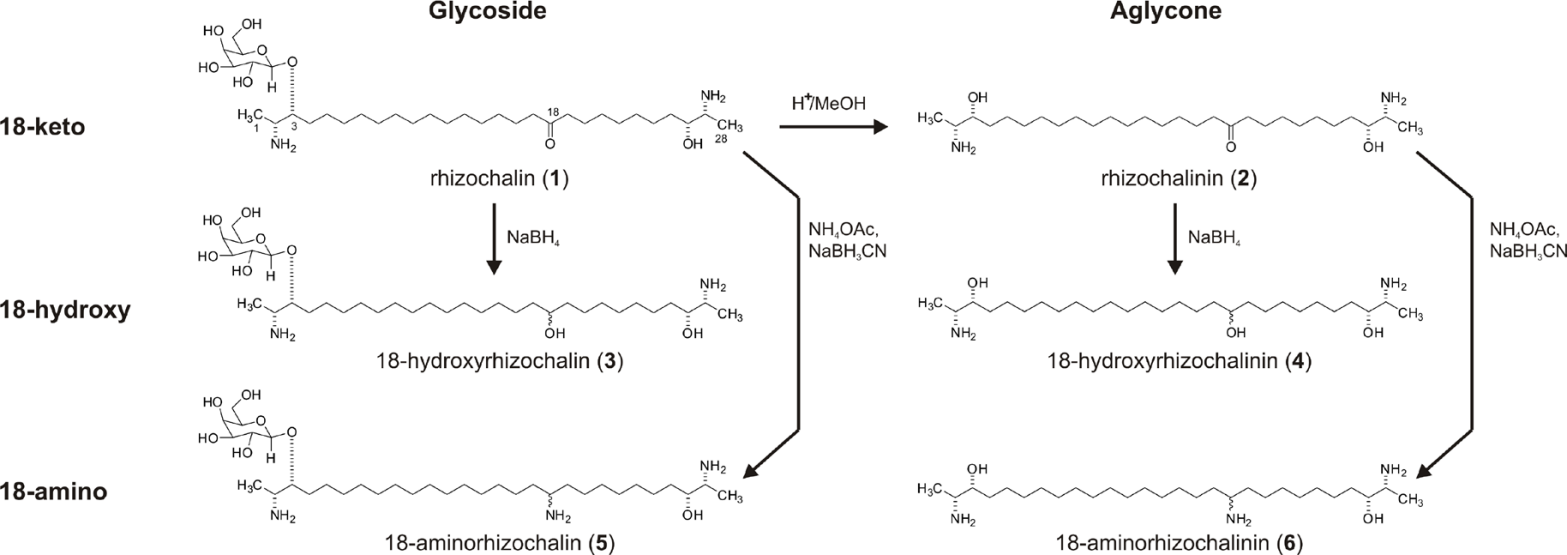
Schema of synthesis of the derivatives (2)–(6) from rhizochalin (1)

Recently, we reported on the activity and the mechanism of action of semi-synthetic marine compound rhizochalinin (1) in human CRPC cells (Figure 1). Rhizochalinin decreases AR-V7 expression accompanied by a resensitization of CRPC cells to enzalutamide [11]. Additionally, the marine compound inhibits pro-survival autophagy [11, 12]. In the current study, we chemically modified the structure of rhizochalin in order to evaluate its structure-activity relationships and optimize anticancer properties. Therefore, the following features of rhizochalinin and its derivates were determined: i) main mechanisms of drug-induced tumor suppression, such as cytotoxic action, antiproliferative activity, and apoptosis induction; ii) inhibition of pro-survival autophagy and iii) inhibition of AR signaling [8, 9].

## RESULTS AND DISCUSSION

### Chemistry

Analyses of structure-activity relationships and further structure optimization are essential steps in the drug development process. For rhizochalin (1) (Figure 1) [13] and similar compounds it has been described that sugar elimination may lead to the increased pro-apoptotic activity [14–16]. The change in the molecule polarity due to an additional reduction of the keto group, or the introduction of nitrogen in the molecule may also affect the biological activity, including pro-apoptotic activity of the drug, as well its effect on autophagy [17]. Thus, taking the chemical properties and stability of the rhizochalin molecule into account we applied the following approaches in order to modify its structure: i) elimination of the sugar residue; ii) reduction of 18-keto group to hydroxyl group; or iii) consecutive reduction and amination of 18-keto group (Figure 1). Rhizochalinin (2) (rhizochalin aglycon) [11, 18] was synthesized from rhizochalin (1) [13] via hydrolysis as reported previously [18] (Figure 1). The four new derivatives (3)–(6) were synthesized from rhizochalin (1) and rhizochalinin (2) via hydrogenation (compounds (3) and (4)) and reductive amination (compounds (5) and (6)). All compounds were purified by reversed-phase flash column chromatography or reversed-phase HPLC and elucidated by spectroscopic methods as follows (for detailed ^1^H NMR data see Supplementary Figure 1):

18-Hydroxyrhizochalin (3). Amorphous solid (61%); ^1^H NMR (CD_3_OD, 500 MHz) δ 4.32 (d, *J* = 7.4 Hz, 1H), 3.79 (d, *J* = 3.1 Hz, 1H), 3.74 (dd, *J* = 8.0, 11.7 Hz, 1H), 3.71 (d, *J* = 4.1, 11.8 Hz, 1H), 3.66 (ddd, *J* = 3.3, 7.3, 9.8 Hz, 1H), 3.53 (dd, *J* = 6.5, 14.0 Hz, 1H), 3.51 (dd, *J* = 7.3, 9.7 Hz, 1H), 3.49 (m, 1H), 3.46 (dd, *J* = 3.1, 9.7 Hz, 1H), 3.39 (m, 1H), 3.15 (p, *J* = 6.7 Hz, 1H), 3.01 (p, *J* = 6.7 Hz, 1H), 1.68 (m, 1H), 1.54 (m, 1H), 1.53 (m, 1H), 1.42 (m, 2H), 1.39 (m, 1H), 1.37 (m, 2H), 1.27–1.30 (br.s, 17H), 1.26 (d, *J* = 6.6 Hz, 3H), 1.22 (d, *J* = 6.6 Hz, 3H); ^13^C NMR (CD_3_OD, 125MHz): δ 104.6 (C-1′), 81.4 (C-3), 77.6 (C-5′), 75.2 (C-3′), 74.5 (C-26), 73.3 (C-2′), 73.1 (C-18), 71.1 (C-4′), 63.6 (C-6′), 53.9 (C-27), 52.5 (C-2), 39.1 (C-17), 39.0 (C-19), 35.3 (C-25), 33.3 (C-4), 31.3-31.5 (C-5-C-16, C-20-C-24), 17.2 (C-28), 16.3 (C-1); HRESIMS: *m/z* 635.52065 [M+H]^+^ (calcd for C_34_H_71_N_2_O_8_, 635.52049).

18-Hydroxyrhizochalinin (4). Amorphous solid (97%); ^1^H NMR (CD_3_OD, 500 MHz) δ 3.50 (m, 1H), 3.43 (m, 2H), 3.08 (sept, *J* = 6.7 Hz, 2H), 1.55 (m, 1H), 1.53 (m, 1H), 1.42 (m, 5H), 1.41 (m, 1H), 1.32 (m, 3H), 1.29 (br.s, 15H), 1.26 (d, *J* = 6.8 Hz, 6H); ^13^C NMR (CD_3_OD, 125MHz): δ 73.8 (C-3, C-26), 73.1 (C-18), 54.1 (C-2, C-27), 39.1 (C-17, C-19), 35.3 (C-25), 26.9 (C-4), 31.2-31.5 (C-6-C-16, C-20-C-23), 27.4 (C-5, C-24), 16.6 (C-1, C-28); HRESIMS: *m/z* 473.4687 [M+H]^+^ (calcd for C_28_H_61_N_2_O_2_, 473.4678).

18-Aminorhizochalin (5). Amorphous solid (79%); ^1^H NMR (CD_3_OD, 500 MHz) δ 4.32 (d, *J* = 7.4 Hz, 1H), 3.79 (d, *J* = 3.1 Hz, 1H), 3.74 (dd, *J* = 8.0, 11.7 Hz, 1H), 3.71 (d, *J* = 4.1, 11.8 Hz, 1H), 3.66 (ddd, *J* = 3.3, 7.3, 9.8 Hz, 1H), 3.54 (m, 1H), 3.51 (dd, *J* = 7.4, 9.8 Hz, 1H), 3.46 (dd, *J* = 3.1, 9.7 Hz, 1H), 3.39 (m, 1H), 3.15 (p, *J* = 6.5 Hz, 1H), 3.07 (p, *J* = 6.5 Hz, 1H), 3.00 (p, *J* = 6.5 Hz, 1H), 1.68 (m, 1H), 1.60 (m, 2H), 1.55 (m, 1H), 1.53 (m, 3H), 1.39 (m, 1H), 1.37 (m, 4H), 1.27–1.30 (br.s, 15H), 1.26 (d, *J* = 6.6 Hz, 3H), 1.21 (d, *J* = 6.8 Hz, 3H); ^13^C NMR (CD_3_OD, 125MHz): δ 104.6 (C-1′), 81.4 (C-3), 77.6 (C-5′), 75.2 (C-3′), 74.6 (C-26), 73.3 (C-2′), 71.1 (C-4′), 63.6 (C-6′), 53.9 (C-27), 53.6 (C-18), 52.6 (C-2), 35.0 (C-17), 35.3 (C-19, C-25), 33.5 (C-4), 31.3-31.5 (C-5-C-15, C-21-C-24), 26.9 (C-16, C-20), 17.3 (C-28), 16.3 (C-1); HRESIMS: *m/z* 634.5357 [M+H]^+^ (calcd for C_34_H_72_N_3_O_7_, 634.5365).

18-Aminorhizochalinin (6). Amorphous solid (97%); ^1^H NMR (CD_3_OD, 500 MHz) δ 3.39 (m, 2H), 3.08 (p, *J* = 6.4 Hz, 1H), 3.01 (m, 2H), 1.60 (m, 2H), 1.55 (m, 2H), 1.54 (m, 2H), 1.39 (m, 2H), 1.27–1.30 (br.s, 15H), 1.22 (d, *J* = 6.8 Hz, 6H); ^13^C NMR (CD_3_OD, 125 MHz): δ 74.5 (C-3, C-26), 53.9 (C-2, C-27), 53.6 (C-18), 35.3 (C-4, C-25), 35.0 (C-17, C-19), 31.1-31.4 (C-6-C-16, C-20-C-23), 17.3 (C-1), 17.2 (C-28); HRESIMS: *m/z* 472.4828 [M+H]^+^ (calcd for C_28_H_62_N_3_O_2_, 472.4837).

### Biology

### Cytotoxicity

**Figure 2:**
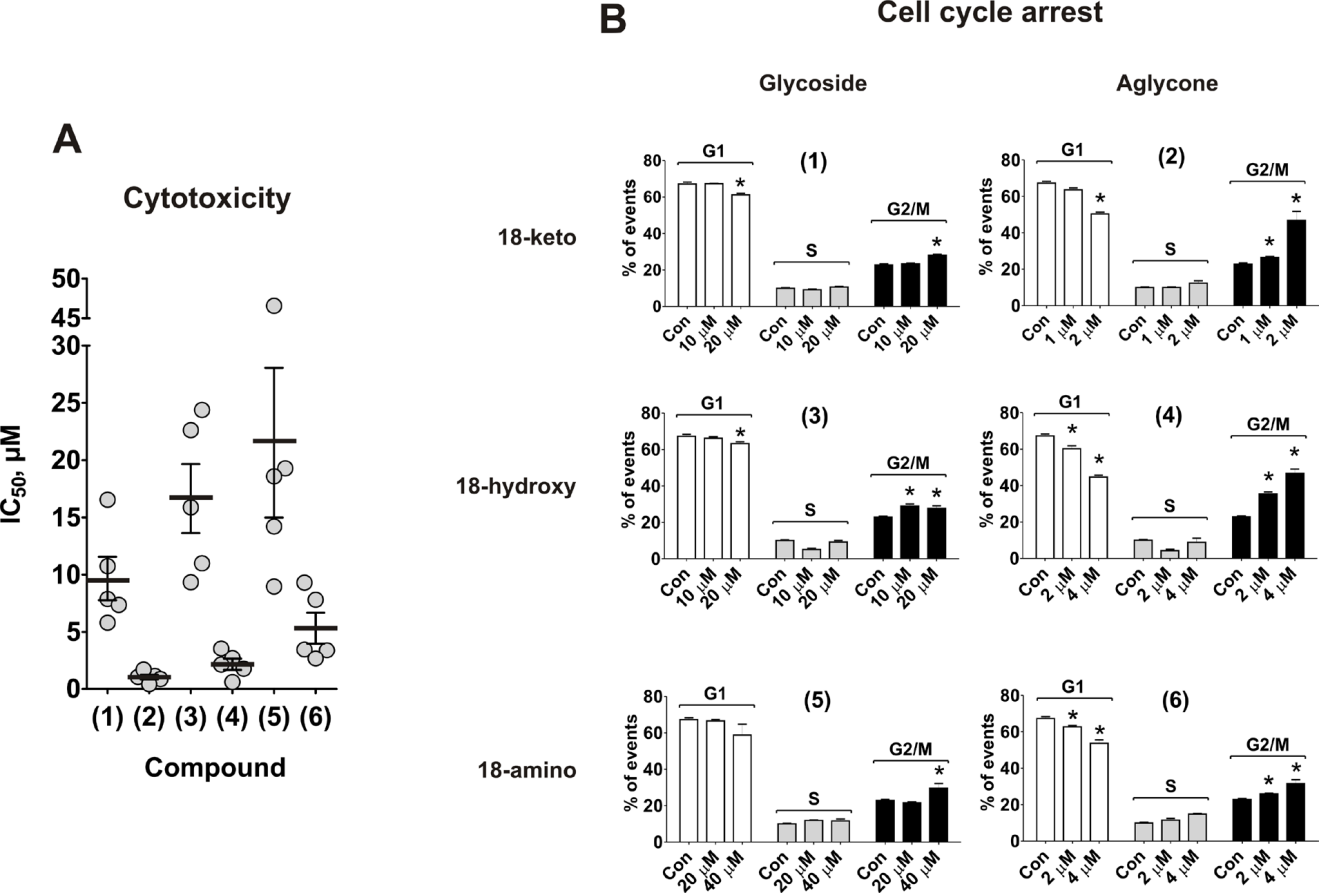
Effect on viability and cell cycle progression of human prostate cancer cells (**A**) MTT assay: each dot represents IC_50_ value (μM) of the compound against certain cancer cell line. The values are equal to those represented in the Table 1. (**B**) PC-3 cells were treated with compounds (1)–(6) for 48 h and the cell cycle phase distribution was quantified using the Cell Quest Pro software. ^*^p < 0.05 (Student's *t*-test).

First, we evaluated the cytotoxic, antiproliferative and pro-apoptotic effects of the substances (1)–(6). The synthesized compounds were tested in human prostate cancer cell lines PC-3, DU145, LNCaP, 22Rv1, and VCaP. All cell lines except LNCaP cells are abiraterone/enzalutamide-resistant due to the absence of AR (PC-3 and DU145) or the presence of AR-V7 (22Rv1 and VCaP). Additionally, PC-3 cells have previously been reported to be docetaxel-resistant [19]. Remarkably, the compounds exhibited cytotoxic activity in all cells lines at micro- or nanomolar concentrations [20, 21] (Table 1). The aglycons (2), (4) and (6) possessed ~10-fold stronger *in vitro* activity when compared to glycosides (1), (3) and (5) (Figure 2A). Additionally, an increase of cytotoxicity was observed in the row 18-amino < 18-hydroxy < 18-keto derivatives (Table 1, Figure 2A).

**Table 1:** Cytotoxic activity of compounds (1)–(6) in human prostate cancer cells after 48 h of treatment

Compound	IC_50_, μM				
	PC-3	DU145	LNCaP	22Rv1	VCaP
rhizochalin (**1**)	16.55 ± 1.37	10.75 ± 1.48	7.88 ± 2.4	7.37 ± 0.69	5.81 ± 0.23
rhizochalinin (**2**)	1.14 ± 0.04	1.05 ± 0.02	1.69 ± 0.38	0.87 ± 0.33	0.42 ± 0.11
18-hydroxyrhizochalin (**3**)	22.62 ± 0.3	24.38 ± 0.38	9.34 ± 0.57	11 ± 1.14	15.89 ± 5.23
18-hydroxyrhizochalinin (**4**)	2.72 ± 0.13	2.13 ± 0.19	3.55 ± 0.45	1.77 ± 0.99	0.61 ± 0.08
18-aminorhizochalin (**5**)	46.57 ± 13.78	19.29 ± 13.08	8.97 ± 2.47	14.21 ± 5.09	18.59 ± 3.46
18-aminorhizochalinin (**6**)	3.39 ± 0.30	7.82 ± 1.12	9.31 ± 2.12	3.46 ± 1.2	2.67 ± 0.52

Data are presented as mean ± SEM.

### Cell cycle progression

To investigate the antiproliferative activity of the compounds we examined their effects on cell cycle progression. Mild, but statistically significant G2/M arrest was observed with a 48 h treatment of all compounds in human cancer PC-3 cells. The effect was most pronounced for aglycones (2) and (4) (Figure 2B). However, in contrast to PC-3 cells, no pronounced cell cycle arrest was observed in 22Rv1 cells (data now shown). This may result from a different genetic background of the cells. Thus, rhizochalin and its derivatives exhibit mainly cytotoxic, rather than antiproliferative activity.

### Induction of apoptosis

**Figure 3:**
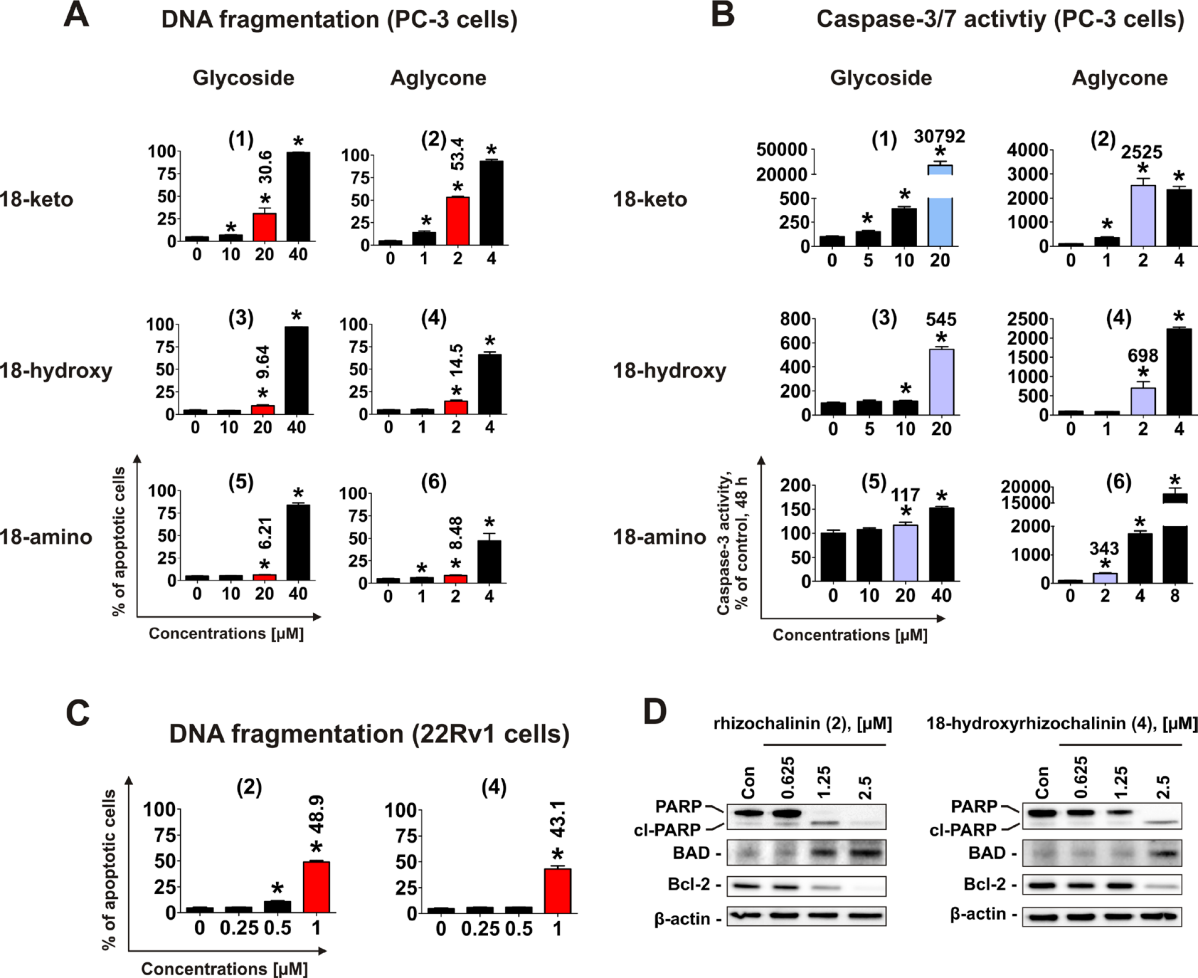
Pro-apoptotic activity of the compounds (1)–(6) in human cancer cells (**A**) Analysis of DNA fragmentation in PC-3 cells treated with compounds (1)–(6) for 48 h at different concentrations. The number of cells with fragmented DNA was assessed with flow cytometry and assumed as the sub-G1 population in cycle analysis. The effects on DNA fragmentation were compared at either 20 μM or 2 μM (red bars on the graphs). (**B**) Analysis of caspase-3/7 activity in PC-3 cells treated with the investigated compounds at different concentrations for 48 h. The effects on caspase-3/7 activity were compared at either 20 μM or 2 μM (blue bars on the graphs). (**C**) FACS analysis of DNA fragmentation in 22Rv1 cells treated with compounds (2) and (4) for 48 h at different concentrations. The effects on DNA fragmentation were compared at 1 μM (red bars on the graphs). (**D**) Analysis of several pro- and anti-apoptotic protein expression in 22Rv1 cells treated with compounds (2) and (4) for 48 h. ^*^p < 0.05 (Student's *t*-test).

Next, effects on induction of apoptosis were examined. Treatment with rhizochalinin and its derivatives increased the sub-G1 population of PC-3 cells, indicating DNA fragmentation (Figure 3A). Additionally, caspase-3/7 activation was detected in cells treated for 48 h (Figure 3B).

To identify the most active derivative, we examined the ability of the different compounds to induce the DNA fragmentation and caspase-3/7 activation. The glucose-containing compounds (1), (3), and (5) are less cytotoxic (i.e. having higher IC_50_s) in comparison with the correspondent aglycons (2), (4), and (6) (Figure 2A, Table 1). Therefore for the further experiments, 20 μM for glycoside derivatives (1), (3) and (5); and 2 μM for aglycons (2), (4) and (6) have been used (Figure 3A, 3B). In accordance with the results of cytotoxicity assay (Figure 2A), 18-keto derivatives exhibited the most pronounced pro-apoptotic activity within the same glycoside or aglycone family, whereas 18-amino derivatives showed the weakest activity. Pro-apoptotic activity of aglycon derivatives (2), (4) and (6) was distinctly more pronounced when compared with glycosides (1), (3) and (5) (Figure 3A, 3B).

Rhizochalinin (2) and 18-hydroxyrhizochalinin (4) were identified to have the strongest cytotoxic effect exerted through the apoptotic mechanism (Figure 3A, 3B). Therefore, we additionally examined the pro-apoptotic effects of these derivatives in castration-resistant AR-V7-positive 22Rv1 cells. Similar to PC-3 cell line, we both compounds induced DNA fragmentation in this prostate cancer cell model (Figure 3C). Interestingly, the pro-apoptotic activity of the compounds (2) and (4) was stronger in 22Rv1 cells when compared to PC-3 cells (Figure 3C). We also examined the effects of the compounds on the expressional levels of several apoptosis-related Bcl-2 family proteins (Figure 3D). Up-regulation of the pro-apoptotic BAD and down-regulation of anti-apoptotic Bcl-2 were detected (Figure 3D), while Bax and Bcl-xL expression were not influenced significantly (data not shown). Finally, another apoptotic marker – PARP cleavage – was detected (Figure 3D). Taken together these results indicate apoptotic character of the drug-induced cells death, which is in line with the caspase-dependent apoptosis induced by rhizochalinin (2) in cancer cells, recently reported by us [11].

### Effect on autophagy

**Figure 4:**
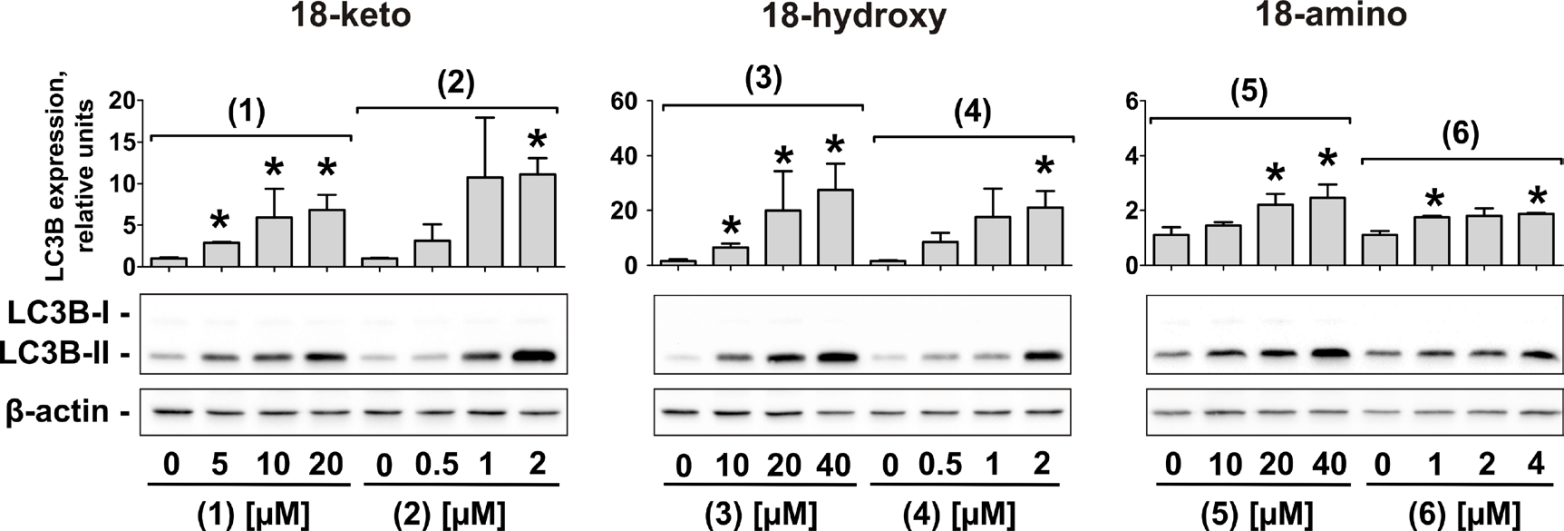
Inhibition of autophagy in human prostate cancer cells under treatment with the compounds (1)–(6) Western blotting analysis of LC3B-I/II proteins in PC-3 cells treated with different concentrations of the compounds (1) – (6) for 48 h. The signal intensity was quantified with Quantity One 4.6 software and normalized to the signal of β-actin. ^*^p < 0.05 (Student's *t*-test).

In human cancer the role of autophagy is discussed controversially [22]. However, it is believed that autophagy is cytotoxic in very early stages of tumor development, but cytoprotective in late stages [22]. Indeed, autophagy leads to selective or non-selective bulk degradation of cellular proteins and organelles. This results in extra nutrients supply and helps cancer cells to overcome stressful conditions (lack of nutrients, chemo- and radiotherapy, etc.), ultimately leading to drug resistance [23, 24]. Thus, autophagy has been identified to be an important mediator of drug-resistance in human CRPC [23, 24]. Rhizochalinin (2) inhibits cytoprotective autophagy in human CRPC cells and thus may be capable to overcome drug resistance [11]. Consequently, we examined the effect of the novel derivatives on autophagy in prostate cancer PC-3 cells [25]. The expression of LC3B-I/II proteins is a well-established marker of autophagy [26]. In this model, an increased LC3B-II level after 48 h-treatment indicates autophagy inhibition, whereas LC3B-II decrease reflects induction of autophagy [27]. All compounds caused an increase of LC3B-II in PC-3 cells (Figure 4). Unlike to the cytotoxic and pro-apoptotic activity, the strongest autophagy inhibitory effect was observed for 18-hydroxy derivatives (3) and (4), and the weakest for 18-amino derivatives (5) and (6) (Figure 4).

### Effect on androgen receptor signaling

AR-V7 expression is accompanied by autoactivation of AR-signaling in prostate cancer cells, mediating resistance to AR-targeting drugs enzalutamide and abiraterone *in vitro* and *in vivo* [6, 20, 21]. In patients, AR-V7 expression is associated with drug resistance and poor prognosis [6]. Thus, treatment approaches which are capable of suppressing AR-V7 expression are of high interest.

**Figure 5:**
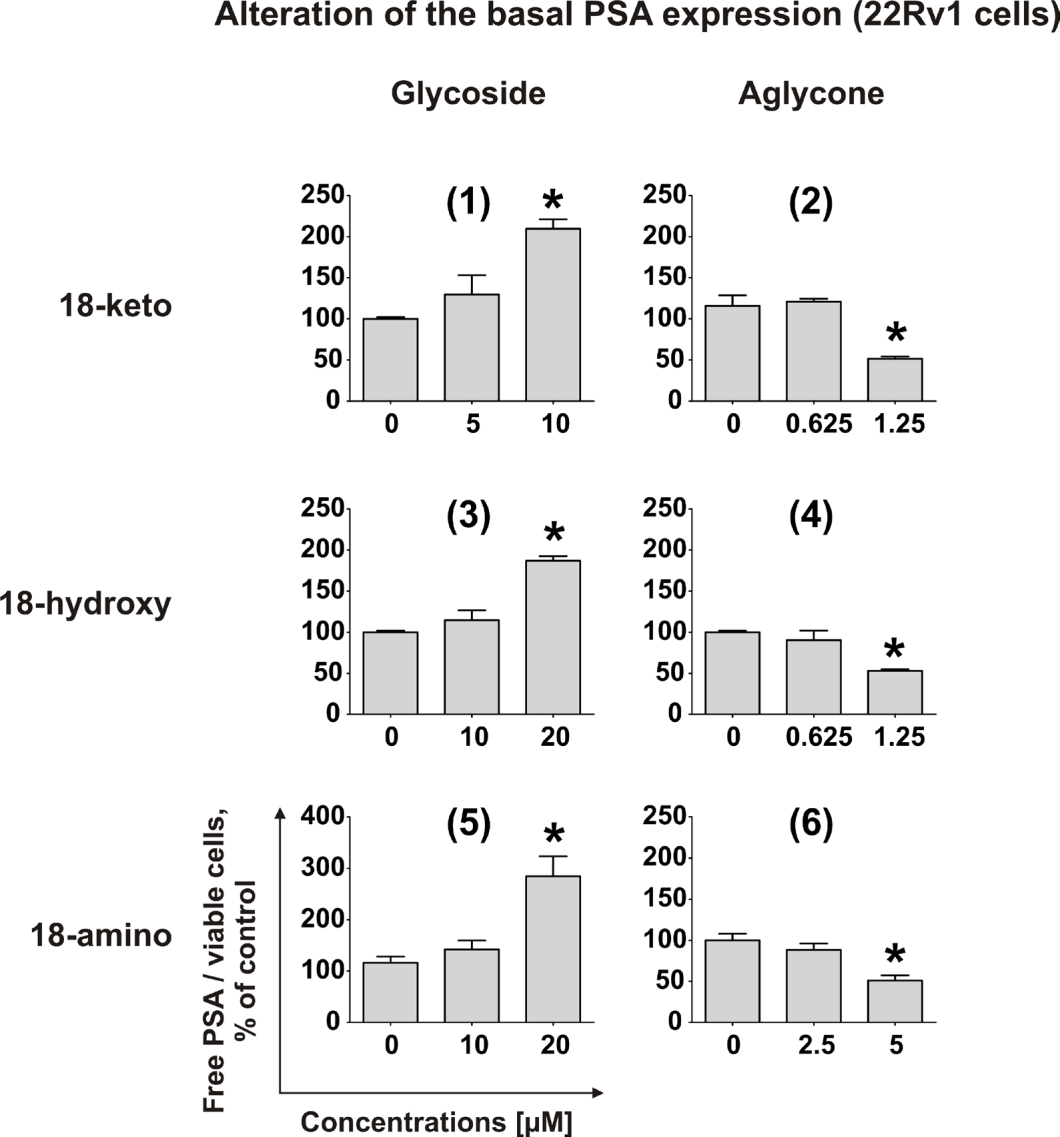
Effect on PSA protein expression Effect of compounds (**1**)–(**6**) on PSA protein expression in 22Rv1 cells. The concentration of PSA in the culture supernatant was analyzed using ELISA and normalized to the number of viable cells. ^*^p < 0.05 (Student's *t*-test).

Recently, we demonstrated that rhizochalinin (2) decreases the basal PSA expression, which is produced in non-DHT-stimulated 22Rv1 cells due to AR-V7 expression [11]. Therefore, we postulated that rhizochalinin suppresses AR-signaling [11]. Consequently, we examined the effect of the newly synthesized derivatives on the same biological target in 22Rv1 cells. The aglycon derivatives (2), (4) and (6) expectably decreased the basal PSA expression, while in contrast the glycosides (1), (3) and (5) surprisingly appeared to increase the PSA production (Figure 5). This finding suggests the particular importance of the sugar elimination for the AR-signaling suppressive properties of the synthesized derivatives.

**Figure 6:**
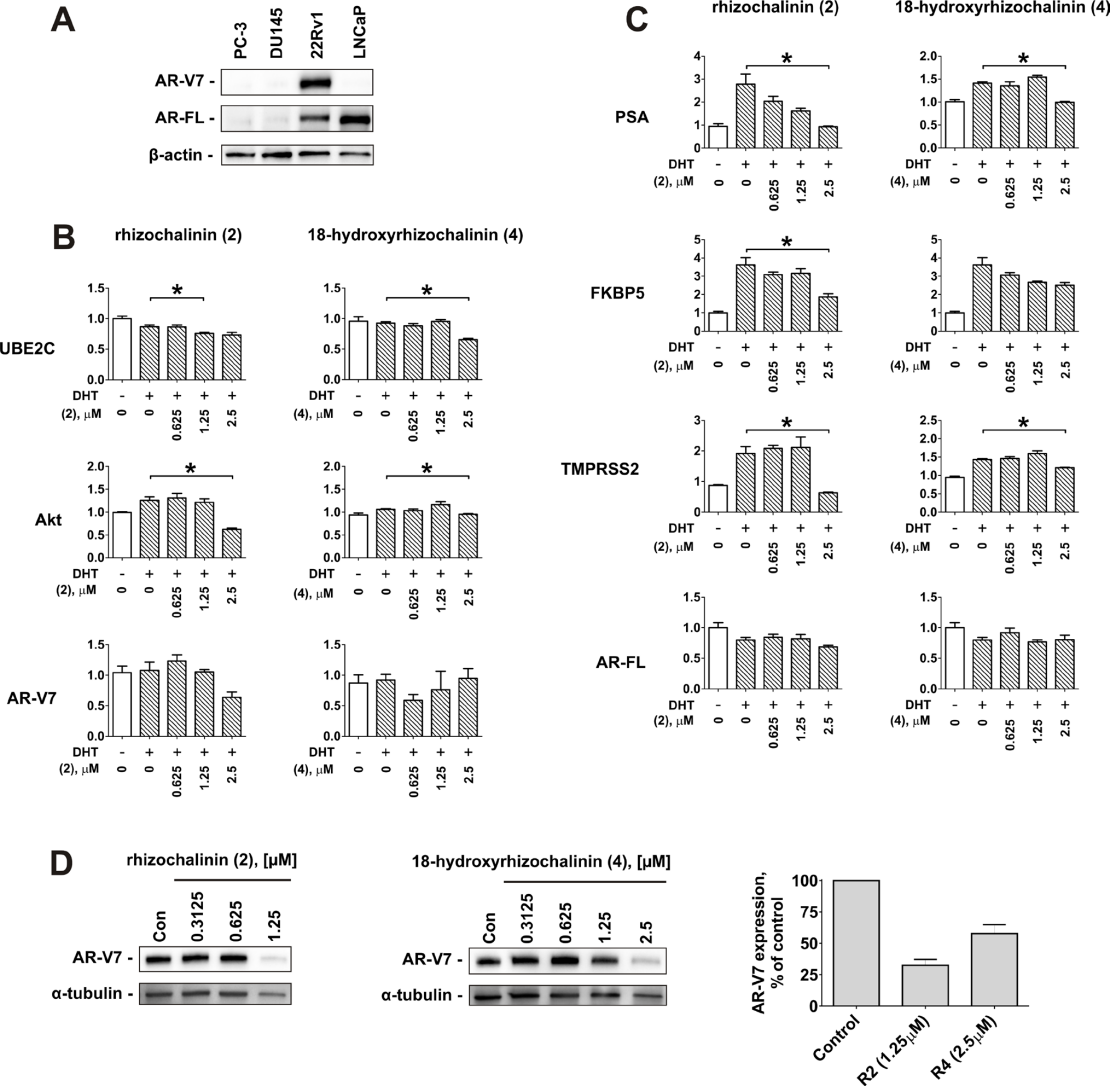
Effect of rhizochalinin (2) and 18-hydroxyrhizochalinin (4) on the expression of different genes, controlled by AR-FL and AR-V7 (**A**) Analysis of AR-V7 and AR-FL protein expression in different human prostate cancer cells lines. (**B**, **C**) mRNA expression levels of the genes controlled by AR-V7 (B) or AR-FL (C). 22Rv1 cells were pre-treated with the indicated concentrations of investigated compounds in 0.1% FBS/RPMI media for 30 min followed by co-treatment with 20 nM DHT for another 24 h. (**D**) Analysis of AR-V7 expression in 22Rv1 cells lines treated with compounds (2) and (4) for 48 h. Signal intensity was quantified with Quantity One 4.6 software and normalized to the signal of α-tubulin. Protein expression was analyzed by Western blotting. mRNA expression was analysed by qPCR. ^*^p < 0.05 (Student's *t*-test).

Taking together the results of the cytotoxic, antiproliferative, pro-apoptotic, autophagy inhibitory, and AR-targeting effects examination, rhizochalinin (2) and 18-hydroxyrhizochalinin (4) have been identified as the most promising derivatives in terms of anticancer activity in human CRPC cells (Table 2). Thus, these two molecules were chosen to further determine their ability to suppress AR-signaling in 22Rv1 cells. 22Rv1 cells are capable of both AR-FL- and AR-V7 expression (Figure 6A) and therefore are resistant to ADT and to the second generation AR-targeted therapies [20, 21]. We used the qPCR technique to examine the effects of derivatives (2) and (4) on the expression of mRNA levels of specific endogenous target genes of AR-V7 (genes AKT1 and UBE2C) (Figure 6B) and AR-FL (genes PSA, TMPRSS2, and FKBP5) (Figure 6C). The AR-FL pathway and the correspondent gene expression were activated by DHT treatment. Similar to the results reported in DHT non-stimulated 22Rv1 cells (see Figure 5), both compounds (2) and (4) significantly suppressed the DHT-stimulated expression of PSA mRNA (Figure 6C), as well as two other AR-FL-controlled genes – TMPRSS2 and FKBP5 (Figure 6C). Even more important, AR-V7-controlled AKT1 and UBE2C were also suppressed in 22Rv1 cells (Figure 6B). Remarkably, (2) and (4) down-regulated AR-V7 protein expression (Figure 6D). This may explain the suppression of AR-V7-dependent signaling (Figure 6D). In contrast, AR-V7 and AR-FL mRNA expression remained unaffected by the investigated drugs and DHT (Figure 6B, 6C). Taken together, rhizochalinin (2) and 18-hydroxyrhizochalinin (4) suppress both, AR-V7- and AR-FL-dependent signaling.

**Table 2:** Summary of biological activities of the compounds (1)–(6) in human prostate cancer cells

Actitiy^*^	Compound					
	(1)	(2)	(3)	(4)	(5)	(6)
Cytotoxicity	++	+++	+	+++	+	++
Anti-proliferative activity	+	++	+	++	+	+
Pro-apoptotic activity	++	+++	+	+++	+	++
Autophagy inhibition	++	++	+++	+++	+	+
AR signaling inhibition	–	+++	–	+++	-	++

^*^“+++” – high activity; “++” – moderate activity; “+” – low activity; “–” – no activity.

## MATERIALS AND METHODS

### Chemistry

The ^1^H and ^13^C NMR spectra were obtained using Bruker Avance III HD-500 spectrometer. Chemical shifts were referenced to the corresponding residual solvent signal (δ_H_ 3.30/δ_C_ 49.60 for CD_3_OD). ESI mass spectra were obtained on an Agilent 6510 Q-TOF LC-MS spectrometer by direct injection in MeOH. HPLC was performed using a Shimadzu Instrument equipped with a differential refractometer RID-10A and YMC-ODS-A (250 × 10 mm) column. TLC was performed on Silica plates (5–17 μm, Sorbfil, Russia), and visualization was accomplished by charring at 150°C with 10% (v/v) H_2_SO_4_ in EtOH.

### General procedure for the isolation of rhizochalin (1) and synthesis of rhizochalinin (2)

Rhizochalin ((1), Figure 1) was isolated from the marine sponge *Rhizochalina incrustata* as described before [13]. Rhizochalinin (rhizochalin aglycon, (2), Figure 1), was synthesized from rhizochalin (1) via hydrolysis as reported previously [18].

### General procedure for the synthesis of hydroxyderivatives (3) and (4)

An excess of NaBH_4_ was added to solution of compound (1) (69 mg) or (2) (31 mg) in 2 mL methanol and the reaction mixture was stirred overnight at room temperature. After completion of the reaction, the reaction mixture was neutralized with acetic acid and the solvent was then evaporated under reduced pressure. The product was purified by YMC gel column flash chromatography using ethanol/water (0 → 60%) mixture as eluent and monitored by thin layer chromatography. The fractions that were eluted with 60% ethanol after reversed phase HPLC (70% ethanol/0.1% trifluoroacetic acid) yielded 18-hydroxyrhizochalin ((3), 42 mg) and 18-hydroxyrhizochalinin ((4), 30 mg) (Figure 1).

### General procedure for the synthesis of aminoderivatives (5) and (6)

An excess of ammonium acetate and solution of (1) (73.6 mg) or (2) (31.0 mg) in 2 mL methanol were mixed. Then, an excess of sodium cyanoborohydride was added and the reaction mixture was stirred overnight at room temperature. After completion of the reaction, the reaction mixture was neutralized with acetic acid and the solvent was evaporated under reduced pressure. The resulting product was further purified either by reversed phase HPLC using 65% ethanol/0.1% trifluoroacetic acid solvent system to afford 18-aminorhizochalin ((5), 58.2 mg) or by YMC gel ODS-A column flash chromatography using ethanol/water (0 → 60%) mixture as eluent to afford 18-aminorhizochalinin ((6), 30 mg).

### Reagents and antibodies

MTT (3-(4,5-dimethylthiazol-2-yl)-2,5-diphenyltetrazolium bromide) reagent, propidium iodide (PI) and dihydrotestosterone (DHT) were purchased from Sigma (Taufkirchen, Germany). For the protein detection the following antibodies were used: anti-AR-FL (Santa Cruz, sc-816, 1:200), anti-AR-V7 (abcam, #198394, 1:1000), anti-BAD (Cell Signaling, #9239, 1:1000), anti-Bax (Cell Signaling, #5023, 1:1000), anti-Bcl-2 (Cell Signaling, #2876, 1:1000), anti-Bcl-xL (Cell Signaling, #2764, 1:1000), anti-LC3B-I/II (Cell Signaling, #2775, 1:1000), anti-rabbit IgG-HRP (Cell Signaling, #7074, 1:5000), anti-mouse IgG-HRP (GE Healthcare, NXA931, 1:10000), anti-β-Actin-HRP (Santa Cruz, sc-1616, 1:200), anti-α-Tubulin (Sigma-Aldrich, T5168, 1:5000).

### Cell lines and culture conditions

The human prostate cancer cells PC-3, DU145, LNCaP, 22Rv1, and VCaP were purchased from ATCC (Manassas, VA, USA). Cell lines were cultured according to the manufacturers' protocols in the correspondent media as has been described before [11]. Cells were continuously kept in culture for a maximum of 3 months, and were routinely checked for contamination with mycoplasma and inspected microscopically for stable phenotype. The previous pharmacokinetics study revealed the effect of rhizochalinin (2) to be maximal after 48 h of treatment [11], therefore for most of experiments 48 h incubation was chosen. The current research was performed according to the Good Laboratory Practice regulations (GLPs).

### *In vitro* MTT-based drug sensitivity assay

The *in vitro* cytotoxicity of the investigated compounds was evaluated using the MTT assay, which was performed as previously described [27, 28]. Cells were incubated with the drugs for 48 h.

### Cell cycle and DNA fragmentation analysis

The cell cycle distribution was analyzed by flow cytometry using PI staining as described before [29]. Cells were pre-incubated overnight in six-well plates (0.2 × 10^6^ cells/well) and then incubated with rhizochalin and its derivates for 48 h. Afterwards cells were trypsinized, fixed, stained with propidium iodide / RNase containing buffer, and analyzed with a BD Bioscience FACS Calibur analyzer (BD Bioscience, Bedford, MA, USA) and BD Bioscience Cell Quest Pro v.5.2.1. software (BD Bioscience, Bedford, MA, USA). Apoptotic cells containing fragmented DNA were detected as a sub-G1 population.

### Caspase-3/7 activity assay

The enzymatic activities of caspase-3 and -7 were measured using Caspase-Glo**^®^** 3/7 Assay Kit (Promega) as described before [30]. In brief, 6000 cells per well were seeded in a 96-well white flat-bottom sterile plate, incubated overnight, and treated with the investigated substances at various concentrations for 48 h. Then the Caspase-Glo**^®^** 3/7 reagent was added to the plates and luminescence was measured using an Infinite F200PRO reader (TECAN, Männedorf, Switzerland). Cell viability was measured using the modified MTT assay as described before [30]: the treated cells were incubated with the MTT reagent for 2 h, the media was removed and the plates were dried, then the formazan crystals were dissolved in DMSO, the solution was transferred to new transparent plates and optical density was measured. The caspase-3/7 activity was normalized to the cell viability at the correspondent drug concentration.

### Analysis of PSA expression

The analysis of free PSA expression was determined as described before [11]. 22Rv1 cells (0.4 × 10^6^ cells/well) were seeded in 6-well plates, incubated overnight and the media was replaced with fresh drug-containing media. After 48 h incubation the aliquots were collected and extracellular human prostate-specific antigen (PSA) was measured in the supernatant by ELISA using the ProStatus^™^ PSA Free-/Total DELFIA^®^ Kit (PerkinElmer, Turku, Finland). PSA concentrations were normalized to the number of viable cells in the correspondent wells, which was measured by trypan blue-based viability assays as described before [28].

### Quantitative real-time PCR (qPCR)

Cells were seeded in Petri dishes (2 × 10^6^ cells per ø 6 cm dish in 5 mL of media for 22Rv1) in 10% FBS/RPMI media and incubated overnight. Then the media was replaced with 5 mL of fresh 0.1% FBS/RPMI media. After 24 h of incubation the cells were treated with the investigated compounds for 30 min in 0.1% FBS/RPMI media followed by co-treatment with 20 nM DHT for another 24 h. Cells were harvested by scratching, pelleted, homogenized using QIAshredder (Cat. # 79654, QIAGEN, Hilden, Germany). Total RNA was isolated using PureLink^®^ RNA Mini Kit (Cat. # 12183018A, Invitrogen, Carlsbad, CA, USA) with the on-column DNA digestion using PureLink^™^ DNase (Cat. # 12185-010, Invitrogen). RNA was diluted up to 30 μL and concentrations were measured. RNA was transcribed into cDNA using Maxima First Strand cDNA Synthesis Kit for RT-qPCR (Cat. # K1642, Thermo Scientific, Vilnius, Lithuania). qPCR was performed using 2X KAPA SYBR FAST qPCR Master Mix Optimized for Roche LightCycler 480 (Cat. # KK4609, KAPA biosystems, Worburn, MA, USA) according to the manufacturer's instructions. 2 pmol of primers and 20 ng of template cDNA were used per one reaction. Expressions of human AR-FL, AR-V7, PSA, AKT, FKBP5, TMPRSS2, UBE2C, and GAPDH genes were analyzed using the specific primers, synthesized by Eurofins MWG-Biotech AG (Ebersberg, Germany). Primer sequences and melting temperatures (Tm) are presented in Supplementary information, Supplementary Table 1. The PCR conditions were 30 sec 95°C, followed by 40 cycles of 15 sec 95°C, 5 sec Tm, and 26 sec 72°C (measurement of fluorescence). Melting curve analysis (10 sec 95°C, 60 sec 65°C and 1 sec 97°C) was performed directly after each PCR run. Relative expression was calculated using the 2^–ΔΔCT^ method. To test statistical significance, data were analyzed by unpaired Student's *t*-tests.

### Protein preparation and western blotting

Preparation of protein extracts and Western blotting were performed as described previously with slight modifications [28]. In brief, 1 × 10^6^ cells/well were seeded in Petri dishes (ø 6 cm, 5 mL/dish) and incubated overnight. Then, the media was substituted with drug-containing media and cells were incubated for 48 h. Cells were harvested, the proteins were extracted using the lysis buffer (1% NP-40 [v/v], 50 mM Tris-HCl (pH 7.6), 0.88% [w/v] NaCl, 0.25% [w/v] sodium cholate, 1 mM Na_3_VO_4_, 0.1 mM PMSF, 1 tablet/10 mL cOmplete Mini EDTA-free EASYpacks protease inhibitors cocktail (Roche, Mannheim, Germany)), subjected to electrophoresis and transferred to PVDF membrane. The membrane was incubated with the primary and secondary antibody. The signal was detected as described before [31]. The signal intensity was quantified with Quantity One 4.6 software (Bio-Rad, Hercules, CA, USA).

### Statistical analysis

The GraphPad Prism software v. 5.01 (GraphPad Prism software Inc., La Jolla, CA, USA) was used to perform the statistical analyses. Data are presented as mean ± SEM (standard error of the mean). The experiments were performed in triplicates and repeated at least three times. The unpaired Student's *t*-test was used to compare the control group and the drug-treated group. Differences were considered to be statistically significant and marked with an asterisk (^*^) if *p* < 0.05.

## CONCLUSIONS

In conclusion, the newly synthesized derivatives of marine natural compound rhizochalin exhibit structure-activity relationships with an increase of cytotoxic properties in the row 18-amino < 18-hydroxy < 18-keto derivatives. In general, aglycones were distinctly more active when compared with glycosides. In fact, sugar elimination was critical for the ability to suppress AR-signaling while all compounds inhibited cytoprotective autophagy.

Based on these results, rhizochalinin (2) and 18-hydroxyrhizochalinin (4) were identified as the most promising derivatives (Table 2). Both compounds suppressed the AR-FL- and AR-V7-depending signaling in human CRPC cells and induced cancer cell apoptosis, and therefore are promising new treatment options for castration-resistant, AR-V7-positive prostate cancer. Remarkably, we have recently shown the efficacy and low toxicity of rhizochalinin (2) *in vivo* in human castration-resistant subcutaneously xenotransplanted prostate cancer cells, whereas 18-hydroxyrhizochalinin (4) still awaits for the *in vivo* examinations [11]. The further structure optimization of the rhizochalin derivatives as well as development of its chemical synthesis from commercially available reagents is currently in progress. The new structures with improved biological and chemical properties as well as the synthesis are to be patented in order to promote further drug development.

## SUPPLEMENTARY MATERIALS FIGURES AND TABLE



## Abbreviations

22Rv1: ADT-resistant AR/AR-V7 positive prostate cancer cell line [21]
AR: Androgen receptor
AR-FL: Full-length androgen receptor
AR-V7: Androgen receptor splice variant 7
ADT: Androgen deprivation therapy
ATCC: American type culture collection
DU145: ADT-resistant AR-negative prostate cancer cell line [32]
LC3B: Microtubule-associated protein light chain 3
LNCaP: ADT-sensitive AR-positive prostate cancer cell line [32]
mCRPC: Metastatic castration resistant prostate cancer
PC-3: ADT-resistant AR-negative prostate cancer cell line [32]
PSA: Prostate specific antigen
VCaP: ADT-resistant AR/AR-V7 positive prostate cancer cell line [21]
