# Supplementary material for: Synthesis and anticancer activity of the derivatives of marine compound rhizochalin in castration resistant prostate cancer
Source: Oncotarget. 2018 Mar 30;9(24):16962–73. doi: 10.18632/oncotarget.24764 (PMC5908298; doi:10.18632/oncotarget.24764)
Supplement: Supplementary file 1 [file oncotarget-09-16962-s001.pdf]

Synthesis and anticancer activity of the derivatives of marine compound rhizochalin in castration resistant prostate cancer

SUPPLEMENTARY MATERIALS

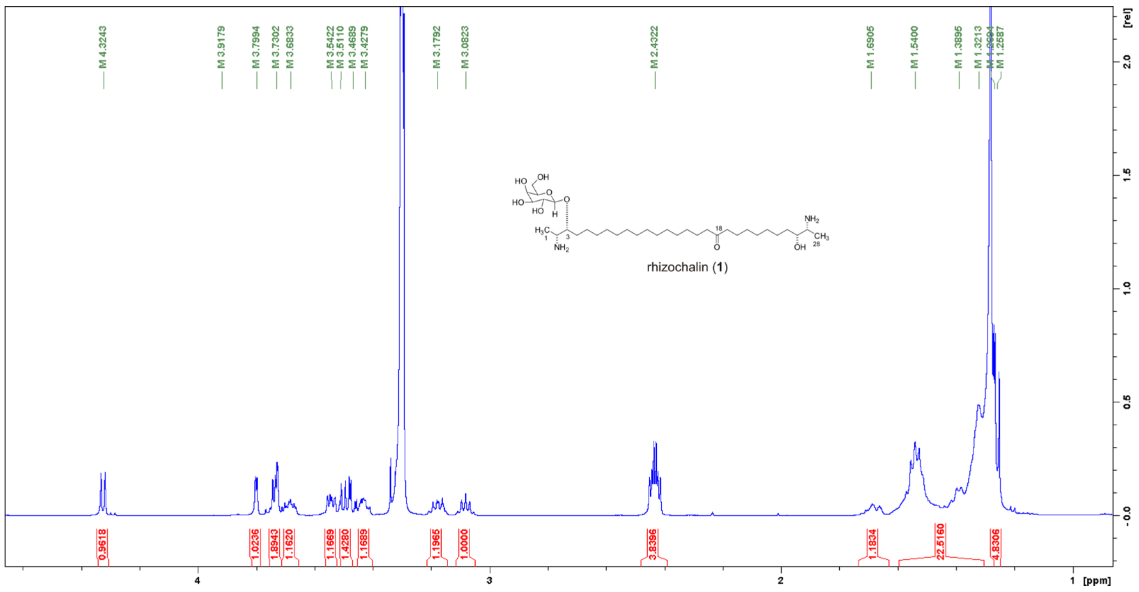

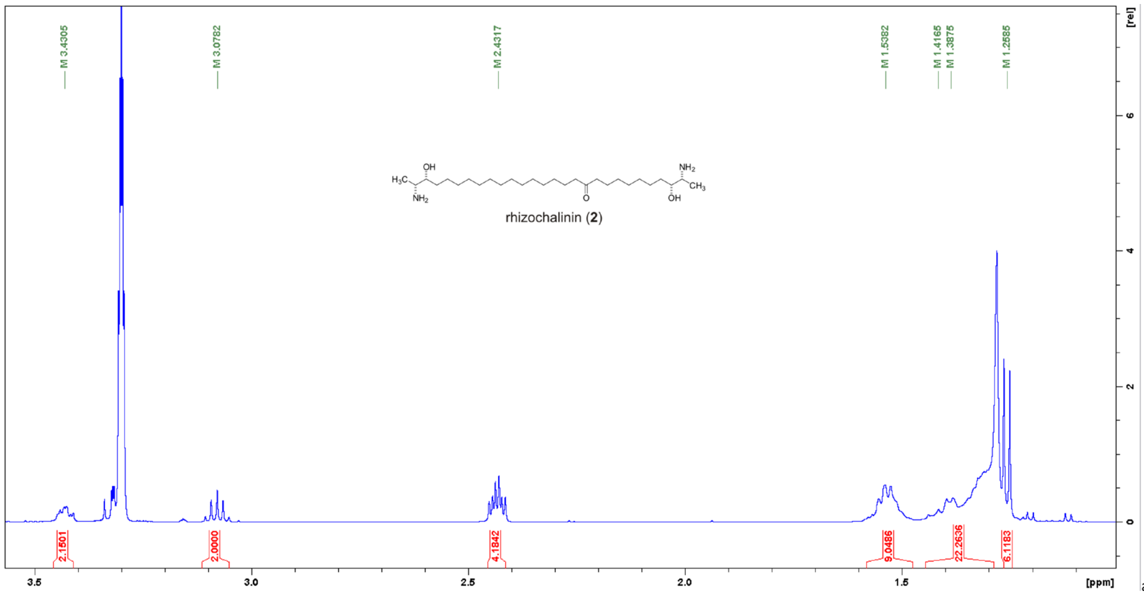

a

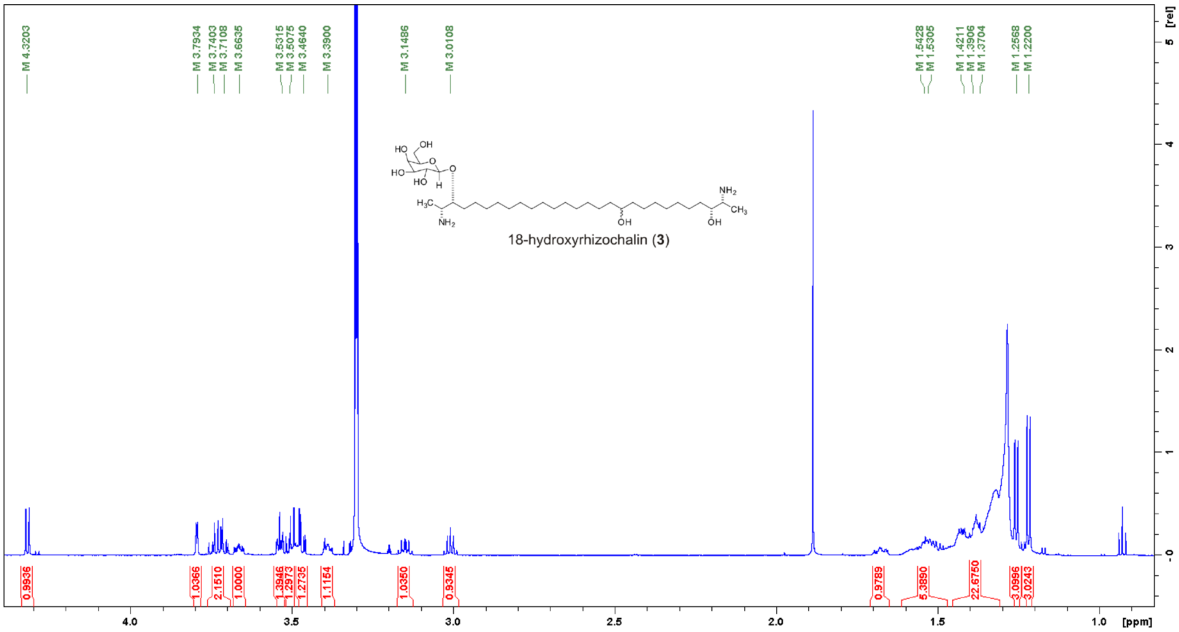
M 4.3203
M 3.7934
M 3.7403
M 3.7108
M 3.6635
M 3.5315
M 3.5075
M 3.4640
M 3.3900
M 3.1486
M 3.0108
M 1.5428
M 1.5305
M 1.4211
M 1.3906
M 1.3704
M 1.2568
M 1.2200
HO
OH
O
O
H
H3C
NH2
OH
18-hydroxyrhizochalin (3)
0.9936
1.0366
2.1510
1.0000
1.3946
1.2973
1.2735
1.1154
1.0350
0.9345
0.9789
5.3890
22.6750
3.0996
3.0243
4.0
3.5
3.0
2.5
2.0
1.5
1.0
[ppm]
[rel]

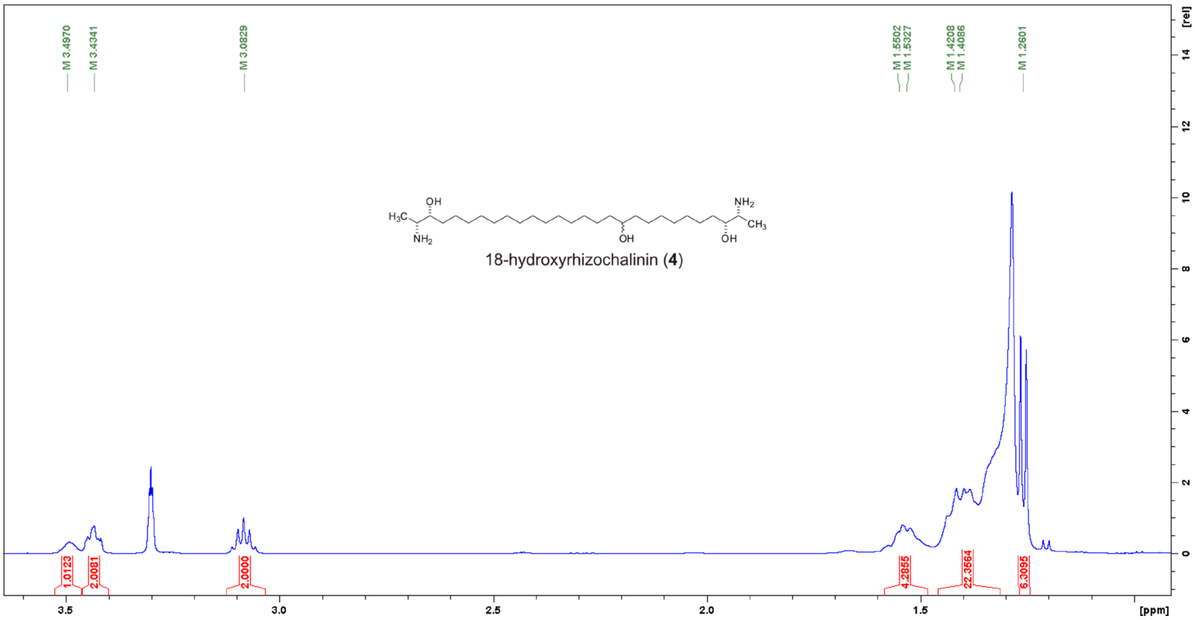
M 3.4970
M 3.4341
M 3.0829
M 1.5502
M 1.5327
M 1.4208
M 1.4086
M 1.2601
OH
H3C
NH2
OH
NH2
CH3
18-hydroxyrhizochalinin (4)
1.0123
2.0081
2.0000
4.2855
22.3564
6.3095
3.5
3.0
2.5
2.0
1.5
[ppm]
[rel]

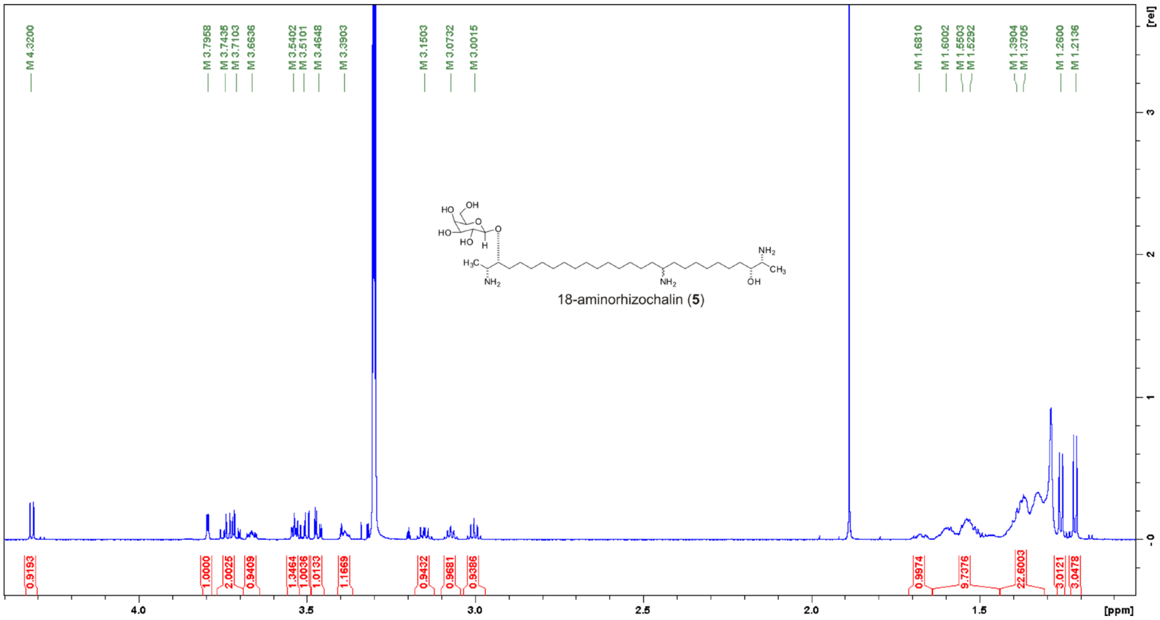

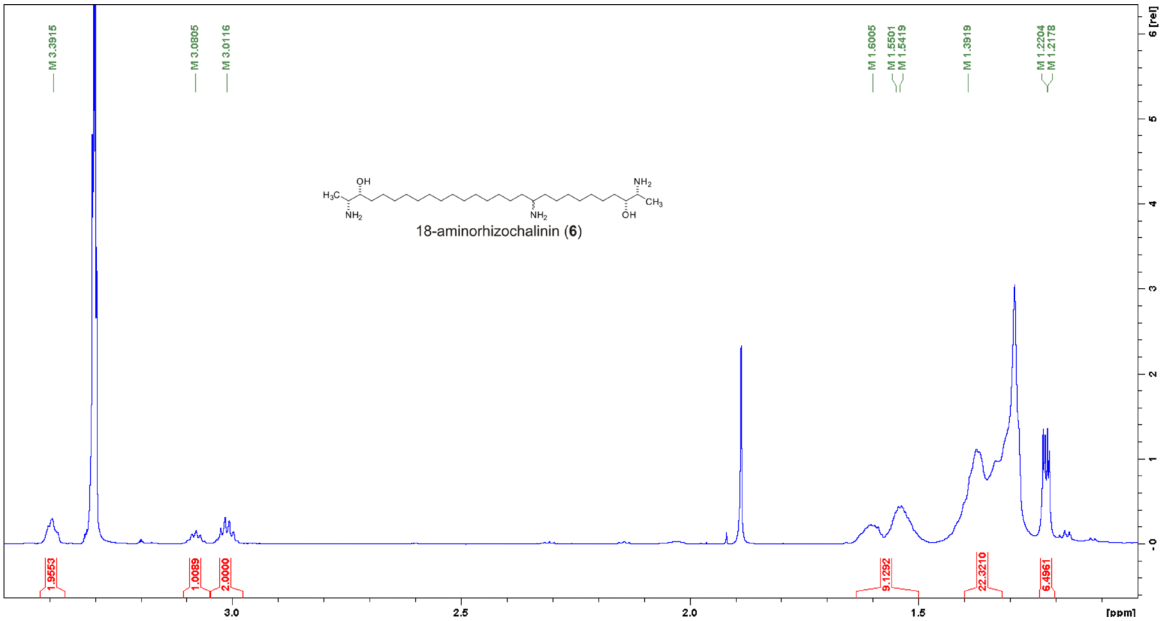

Supplementary Figure 1: ^{1}H NMR spectra of the compounds (1)–(6).

Supplementary Table 1: List of primers used

Primer name	Tm [°C]	Primer sequence
AR-V7 forward	70	5′-CCA TCT TGT CGT CTT CGG AAA TGT TA-3′
AR-V7 reverse		5′-TTT GAA TGA GGC AAG TCA GCC TTT CT-3′
AR-FL forward	61	5′-CAGTGGATGGGCTGAAAAAT-3′
AR-FL reverse		5′-GGAGCTTGGTGAGCTGGTAG-3′
PSA forward	64	5′-CAT CAG GAA CAA AAG CGT GA-3′
PSA reverse		5′-ATA TCG TAG AGC GGG TGT GG-3′
FKBP5 forward	61	5′-AGGAGGGAAGAGTCCCAGTG-3′
FKBP5 reverse		5′-TGGGAAGCTACTGGTTTTGC-3′
TMPRSS2 forward	64	5′-CAGGAGTGTACGGGAATGTGATGGT-3′
TMPRSS2 reverse		5′-GATTAGCCGTCTGCCCTCATTTGT-3′
UBE2C forward	57	5′-TGGTCTGCCCTGTATGATGT-3′
UBE2C reverse		5′-AAAAGCTGTGGGGTTTTTCC-3′
AKT1 forward	62	5′-TGGACTACCTGCACTCGGAGAA-3′
AKT1 reverse		5′-GTGCCGCAAAAGGTCTTCATGG-3′
GAPDH forward	56	5′-TGC ACC ACC AAC TGC TTA-3′
GAPDH reverse		5′-GAG GCA GGG ATG ATG TTC-3′
